# Diagnostic yield and novel candidate genes for neurodevelopmental disorders by exome sequencing in an unselected cohort with microcephaly

**DOI:** 10.1186/s12864-023-09505-z

**Published:** 2023-07-27

**Authors:** Chunli Wang, Wei Zhou, Luyan Zhang, Luhan Fu, Wei Shi, Yan Qing, Fen Lu, Jian Tang, Xiucheng Gao, Aihua Zhang, Zhanjun Jia, Yue Zhang, Xiaoke Zhao, Bixia Zheng

**Affiliations:** 1 Nanjing Key Laboratory of Pediatrics, Children’s Hospital of Nanjing Medical University, Nanjing, China; 2 Department of Neurosurgery, Children’s Hospital of Nanjing Medical University, Nanjing, China; 3 Department of Rehabilitation Medicine, Children’s Hospital of Nanjing Medical University, Nanjing, China; 4 Department of Radiology, Children’s Hospital of Nanjing Medical University, Nanjing, China; 5 Jiangsu Key Laboratory of Pediatrics, Nanjing Medical University, Nanjing, China

**Keywords:** Microcephaly, Neurodevelopmental disorders, Novel candidates, Whole exome sequencing

## Abstract

**Objectives:**

Microcephaly is caused by reduced brain volume and most usually associated with a variety of neurodevelopmental disorders (NDDs). To provide an overview of the diagnostic yield of whole exome sequencing (WES) and promote novel candidates in genetically unsolved families, we studied the clinical and genetic landscape of an unselected Chinese cohort of patients with microcephaly.

**Methods:**

We performed WES in an unselected cohort of 103 NDDs patients with microcephaly as one of the features. Full evaluation of potential novel candidate genes was applied in genetically undiagnosed families. Functional validations of selected variants were conducted in cultured cells. To augment the discovery of novel candidates, we queried our genomic sequencing data repository for additional likely disease-causing variants in the identified candidate genes.

**Results:**

In 65 families (63.1%), causative sequence variants (SVs) and clinically relevant copy number variants (CNVs) with a pathogenic or likely pathogenic (P/LP) level were identified. By incorporating coverage analysis to WES, a pathogenic or likely pathogenic CNV was detected in 15 families (16/103, 15.5%). In another eight families (8/103, 7.8%), we identified variants in newly reported gene (*CCND2*) and potential novel neurodevelopmental disorders /microcephaly candidate genes, which involved in cell cycle and division (*PWP2*, *CCND2*), CDC42/RAC signaling related actin cytoskeletal organization (*DOCK9*, *RHOF*), neurogenesis (*ELAVL3*, *PPP1R9B*, *KCNH3*) and transcription regulation (*IRF2BP1*). By looking into our data repository of 5066 families with NDDs, we identified additional two cases with variants in *DOCK9* and *PPP1R9B*, respectively.

**Conclusion:**

Our results expand the morbid genome of monogenic neurodevelopmental disorders and support the adoption of WES as a first-tier test for individuals with microcephaly.

**Supplementary Information:**

The online version contains supplementary material available at 10.1186/s12864-023-09505-z.

## Background

Microcephaly is clinically defined by an occipitalfrontal circumference (OFC) of > 2 SDs below the mean for age, sex and ethnicity, which is frequently associated with a variety of neurodevelopmental disorders [1]. The underlying etiology is highly heterogeneous and can be intrauterine exposure to infections, toxin or teratogen exposure, and more commonly genetic syndromes [2, 3]. It is classified into primary microcephaly (PM) when it presents at birth, and microcephaly developing after birth is often termed as secondary microcephaly (SM) [4, 5]. PM has been shown most usually due to early impaired neocortical development. Known monogenic causes for PM have been implicated in several cellular processes during brain development, such as centriole biogenesis [6], cytokinesis [7], centromere and kinetochore function [8], DNA replication and repair [9].

Recently, several studies have been made to decipher the underlying genetic causes of microcephaly. The contribution of copy number variants (CNV) and monogenic causes to microcephaly is ranged in different cohort from ~ 40%-80% [10–12]. The advent of genomic sequencing, and particularly the whole exome sequencing (WES), has led to a widespread increase in the clinical diagnostic yield and new Mendelian genes discovery associated with congenital microcephaly, and neurodevelopmental disorders with microcephaly [10, 11]. In this study, we applied WES to a cohort of 103 patients with neurodevelopmental disorders from 103 non-consanguineous families with microcephaly as one of the features. Firstly, we evaluate the clinical diagnostic yield of genomic sequencing on unselected microcephaly individuals by incorporating copy number variations analysis to WES. Furthermore, full evaluation of potential novel candidate genes was applied in genetically undiagnosed families. We describe the new candidate genes from the following aspects: I) expressional trajectories during mammalian cerebral cortex development by analyzing published single-cell mRNA sequencing data; II) available neurologic phenotype from existing animal models (zebrafish or mice); III) involvement of known biological processes perturbed in neurodevelopment. To augment the discovery of novel candidates, we assessed the candidacies for additional families based on our data repository of WES data from 5066 families with NDDs.

## Results

### Cohort characteristics

Totally, 103 unrelated patients with microcephaly of unknown etiology were enrolled. The female to male ratio was 55 to 48. The median age at latest investigation was 1 year 3 months old, ranging from 1 months old to 9-year-olds. PM and SM were determined in 53 (51.5%) and 36 (35.0%) patients, respectively (Table 1). In the other 14 (13.6%) patients, the onset of microcephaly could not be determined. Apart from microcephaly, varying degrees of different neurological signs were reported, among which developmental delay (GDD, ID) and cerebral MRI abnormalities represented the most common associated features (Table 1). There were additional features, such as dystonia or movement disorder, seizures, short stature/growth delay, and heart /urogenital malformations. An overview of the phenotypic spectrum of this cohort are summarized in Table 1.

**Table 1:** Summary of the main clinical characteristics in our cohort of 103 patients with microcephaly

Features	Number of cases
Sex	55 females, 48 males
Age	Median 1.25 years old
Microcephaly	103/103, 100%
Primary	53, 51.5%
Secondary	36, 35.0%
Unknown onset	14, 13.6%
GDD	92, 89.3%
ID (≥ 5y)^a^	14/23, 13.5%
ASD	6, 5.8%
ADHD	12, 11.7%
Dystonia or movement disorder	21, 20.4%
Epilepsy/seizures	10, 9.7%
Abnormal cerebral MRI	32, 31%
Hearing problems	4, 3.9%
Facial dysmorphism	57, 55.3%
Abnormality of the eye	14, 13.6%
Short stature or growth delay	32, 31.1%
Heart defect/Urogenital anomalies	33, 32.0%

*Abbreviations*: *ASD* Autism spectrum disorder, *ADHD* Attention deficit and hyperactivity disorder, *GDD* Global developmental delay, *ID* Intellectual disability

^a^23/103 patients were above the age of 5 years at last investigation and were evaluated for severity of ID using Wechsler intelligence scale

### Diagnostic yield

**Fig. 1:**
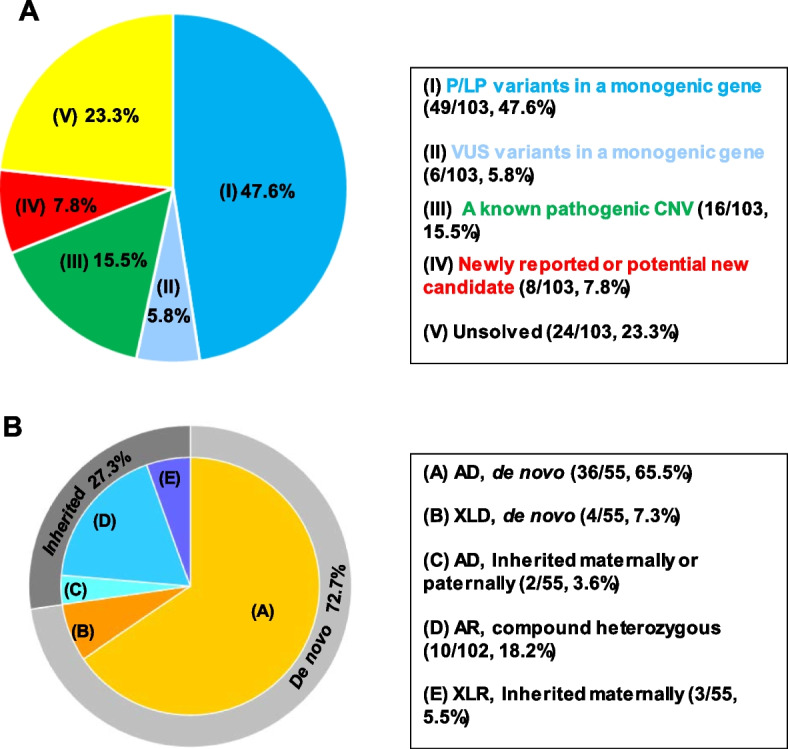
Molecular diagnostic yield and monogenic variants inheritance in 103 families with primary or secondary microcephaly. **A**, Number and percentage of 103 patients in which P/LP level variants in a monogenic cause (49/103, 47.6%), VUS level variants in a monogenic cause (6/103, 5.8%), a known pathogenic CNV (15.5%) or a newly reported or potential new candidate gene (7.8%) was detected by whole-exome sequencing. Blue color denotes that a variant in genes with established disease phenotypes in humans was detected. Green color denotes that a pathogenic or likely pathogenic CNV was detected. Red was chosen if one highly potential novel gene was detected in the family. Yellow indicates no causative or candidate variants were detected. VUS, variant of uncertain significance; LP, likely pathogenic; P, pathogenic. **B**, Inheritance confirmation of sequence variants (SVs) in 55 patients with a known monogenic cause. Segregation validation revealed 36/55 (65.5%) were diagnosed with autosomal dominant (AD) due to de novo variants, 4/55 (7.3%) with X-linked dominant (XLD) de novo, 2/55 (3.6%) inherited AD form, 10/102 (18.2%) with autosomal recessive form, 3/55(5.5%) with X-linked recessive mode (XLR)

In 65 families (63.1%), causative SVs and clinically relevant CNVs with a pathogenic or likely pathogenic (P/LP) level were identified as indicated in Fig. 1A. In another 6 families, a variant of uncertain significance (VUS) level was reported (Fig. 1A). Totally, an SV was detected in 55 patients (55/103, 53.4%) owing to 44 genes with established neurodevelopmental phenotypes in humans (Supplementary Table S1). In particular, 10 of them were diagnosed with autosomal recessive, 38 with autosomal dominant, and 7 with X-linked forms (Fig. 1B). Noteworthy, segregation confirmation revealed a high percentage (72.7%) of de novo variants in our cohort (Fig. 1B).

By incorporating coverage analysis to WES, a pathogenic or likely pathogenic CNV was detected in 15 families (16/103, 15.5%). In another eight families (8/103, 7.8%), eight convincing candidate genes that were not previously reported in regard to neurodevelopmental disorders or microcephaly were identified (Fig. 1A).

### Expression, annotation, and functional network of mutated genes

**Fig. 2:**
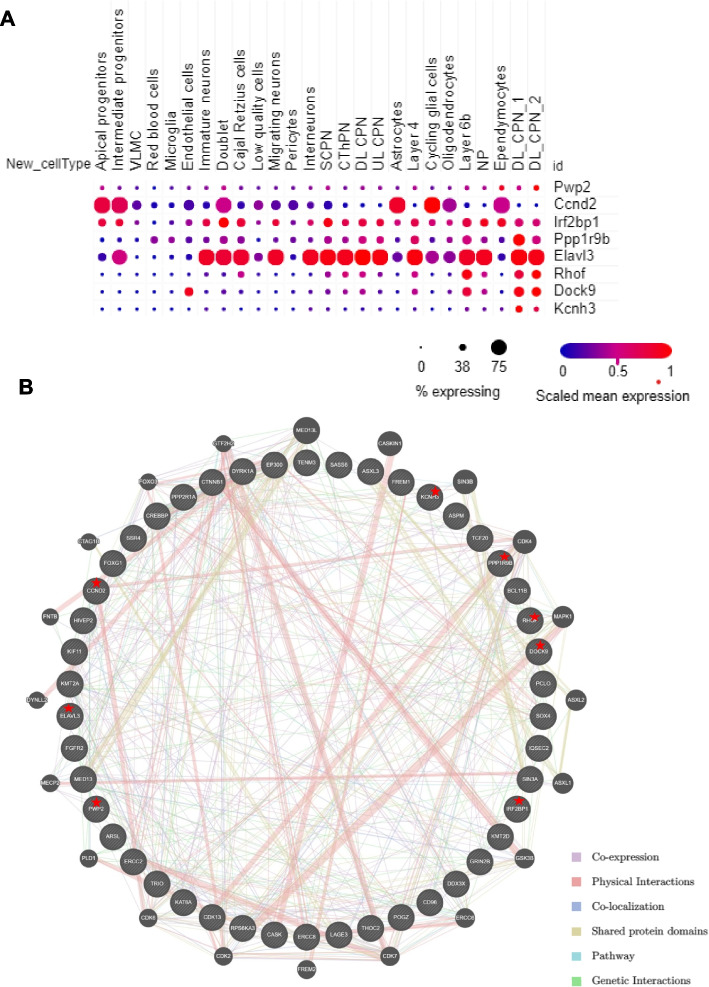
The expressional trajectories in developing cerebral cortex and functional network of known and candidate genes in this cohort. **A**, Expressional trajectories of 8 candidate genes identified in our cohort in neocortical cell types during mouse cortical development. Data was generated from single cell RNA-Seq data provided by Paola Arlotta (Nature 595:554–559,2021). CPN, callosal projection neurons; CThPN, corticothalamic projection neurons; SCPN, subcerebral projection neurons; VLMC, vascular and leptomeningeal cells. **B**, Functional network from GeneMANIA showing the interaction among the mutated known genes and eight candidate genes (highlighted with red star) in our cohort. Analysis was based on co-expression (purple edges), physical interactions (red edges), co-localization (dark blue), shared protein domains (light yellow edges), pathway (light blue edges), and genetic interactions (green edges)

We identified likely deleterious variants affecting one newly reported gene (*CCND2*) and seven different high-level candidate genes in eight (7.8%) patients without P/LP variants or VUS in established disease genes (Table 2). Four of them (*PWP2*, *DOCK9*, *RHOF*, *KCNH3*) were affected by biallelic variants, and four (*CCND2*, *IRF2BP1*, *PPP1R9B*, *ELAVL3*) by a *de novo* variant (Table 2 and Supplementary Fig. S2). We first evaluated the 44 mutated monogenic known genes in our cohort for distinct temporal-spatial single cell mRNA expression pattern, by searching published single-cell transcriptomic data of the developing mouse neocortex and human brain organoids [13, 14]. The eight novel genes or candidates showed expression levels similar to or higher than the expression levels of the 44 mutated known genes in mammalian developing cerebral cortex (Fig. 2A and Supplementary Fig. S1).

**Table 2:** Cases with variants in candidate genes, along with available evidence from the KO phenotype and literature to support candidacy

Family ID	Gene	Genotype	Clinical synopsis	Gene Description	Constraint scores^a^	KO phenotype^b^	Gene function
NJ1050	*PWP2*	CompHet: c.1457G > A;p.Trp486Ter / c.1979G > A;p.Arg660Gln	PM, brain atrophy, GDD, short stature	Small subunit processome component;	Z = 0.32 pLI = 0	A zebrafish mutant with a recessive lethal variant in pwp2h, exhibiting smaller eyes, a smaller, misshapen head	Ribosome biogenesis and cell cycle regulation
NJ3099	*CCND2*	De novo: c.505C > T;p.Gln169Ter	PM, growth retardation, hypothyroidism, dysmorphic features	G1/S-specific cyclin-D2	Z = 2.13 pLI = 0.99	Mutants also show decreased cerebellar granule cell and stellate neuron populations	Regulation of the cell-cycle during G(1)/S transition
NJ233	*DOCK9*	CompHet: c.5887C > T;p.Arg1963Trp/ c.4849 T > C;p.Trp1617Arg	Microcephaly, simplified gyral pattern, epilepsy, GDD, esotropia, dysmorphic features	Dedicator of cytokinesis protein 9	Z = 3.10 pLI = 1.00	ND	Regulation of dendrite growth in hippocampal neurons through activation of CDC42
NJ2639	*RHOF*	CompHet: c.142 T > C;p.Tyr48His/ c.343C > A;p.Pro115Thr	Microcephaly, GDD, hypotonia	Rho-related GTP-binding protein RhoF	Z = 0.87 pLI = 0	ND	Functions cooperatively with CDC42 and Rac to generate additional structures, increasing the diversity of actin- based morphology
NJ463	*ELAVL3*	De novo: c.889G > A;p.Glu297Lys	PM, severe cortical dysplasia, malnutrition, feeding difficulties	ELAV-like protein 3	Z = 3.00 pLI = 0.77	Mice heterozygous for the allele exhibit abnormal brain wave pattern and spike wave discharge	Neuronal differentiation and maintenance
NJ2544	*PPP1R9B*	De novo: c.1610C > T;p.Ala537Val	Microcephaly, GDD, erythema, atrial septal defect with pseudoventricular aneurysm, dysmorphic features	Neurabin-2	Z = 3.02 pLI = 1.00	Homozygotes mutants exhibited abnormal glutamatergic synaptic transmission and increased dendritic spine density	Modulates excitatory synaptic transmission and dendritic spine morphology
NJ316	*IRF2BP1*	De novo: c.136G > T;p.Glu46Ter	Neonatal-onset microcephaly, epilepsy, hypotonia and GDD	Interferon regulatory factor 2-binding protein 1	Z = 3.34 pLI = 0.99	ND	Transcription corepressor activity and ubiquitin protein ligase activity
NJ3479	*KCNH3*	CompHet: c.961C > T ;p.His321Tyr/ c.2812_2813del; p.Leu938ValfsTer73	GDD, PM, motor developmental delay, growth retardation, dysmorphic features	Potassium Voltage-Gated Channel Subfamily H Member 3	Z = 3.85 pLI = 1.00	Mice homozygous for a knock-out allele exhibit neuron hyperexcitability and epilepsy	A voltage-gated potassium channel alpha subunit predominantly expressed in the forebrain; FOXG1-target gene

^a^Constraint scores utilizing a larger dataset of ∼141,000 exomes and genomes from the Genome Aggregation Database (gnomAD); Z, missense Z scores; pLI, probability of being loss-of-function intolerant

^b^Neurological phenotype data from Mouse Genome Informatics or published zebrafish model; *KO* Knockout, *ND* Not described, *GDD* Global developmental delay, *PM* Primary microcephaly

Biological functional annotation revealed that the novel and known mutated genes in our cohort converge on transcription and transcription regulation, host-virus interaction, cell cycle and division, chromosome partition, biological rhythms and neurogenesis (Supplementary Fig. S1). Network analysis using GeneMania revealed that the eight candidate genes interact with each other and with the mutated known genes in our cohort by means of co-expression (49.62%), physical interactions (28.08%), co-localization (13.79%), shared protein domains (4.05%) and genetic interactions (1.79%) (Fig. 2B).

### Variants in candidate and novel gene involved in cell cycle and cell division (*PWP2* and *CCND2*)

**Fig. 3:**
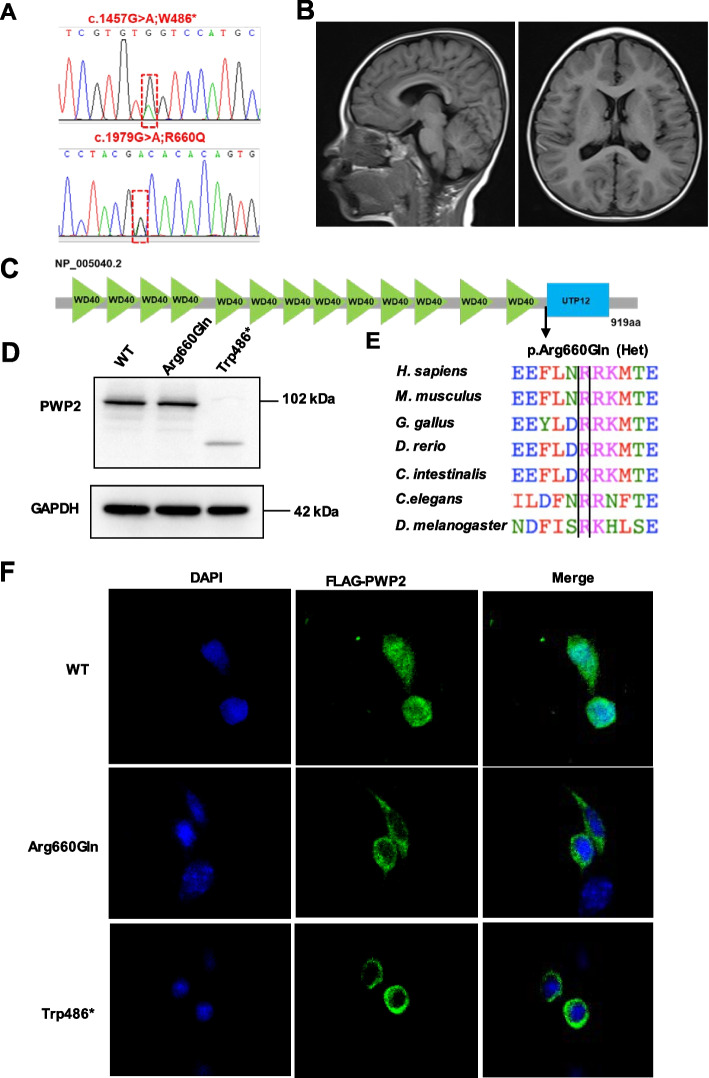
WES identified compound heterozygous variants in *PWP2* in a male patient with primary microcephaly and global developmental delay. **A**, Sequencing chromatograms of the compound heterozygous variants c.1457G > A;p.W486Ter and c.1979G > A;p.R660Q of the *PWP2* gene. **B**, Brain MRI of patient NJ1050 showing cortical atrophy. **C**, Protein domain content of *PWP2* protein. The p.R660Q missense change detected in the family is mapped to the WD40 domain. **D**, Transient overexpression of N-terminally Flag-tagged cDNA constructs modeling the wild-type allele and two independent *PWP2* variants (p.Arg660Gln and p.Trp486Ter) in HEK293 cells. The p.Trp486Ter variant produced a lower mount band with decreased expression. **E**, Multiple sequence alignment of the *PWP2* protein region flanking residue Arg660. The Arg660 is well conserved from Homo sapiens to Drosophila melanogaster. **F**, HEK293 cells was transfected with N-terminal Flag-tagged wild-type PWP2 or mutants. Cells were imaged by confocal microscopy. Representative images of Flag-tagged protein and DAPI localization are shown, revealing that wild-type PWP2 localizes to nuclear, while patient mutants mis-localized diffusely to the cytoplasm

In patient NJ1050, by trio WES we identified compound heterozygous variants (c.1457G>A;p.Trp486Ter and c.1979G>A;p.Arg660Gln) in *PWP2* in a 2 years old boy, who had presented with primary microcephaly (at birth 30.1cm,-3.27SD, at 1 years old 42.1cm, -4.52SD), short stature, global developmental delay, cerebral paralysis and cortical atrophy on MRI (Table 2, Fig. 3A-B). The nonsense p.Trp486Ter variant was absent from the control database gnomAD. The second allele variant p.Arg660Gln occurred 14 times heterozygously (0/14/282772) in the gnomAD database and yielded predominantly deleterious prediction scores by three algorithms (PolyPhen-2, MutationTaster and SIFT). As shown in Fig. 3C*,* the Arg660 residue in PWP2 is well conserved from *Homo sapiens* to *D. melanogaster* (Fig. 3E). We transient overexpressed N-terminally Flag-tagged cDNA constructs modeling the wild-type allele and two independent *PWP2* variants in HEK293 cells*.* As shown in Fig. 3D, the p.Trp486* variant produced a lower mount band with decreased expression. Previous studies have shown that PWP2 is localized in the area of the nucleolus involving in ribosome biogenesis and cell cycle progression [15]. Consistent with this, wildtype PWP2 localized to discrete nuclear region, while PWP2 bearing the case associated variants (p.Arg660Gln and p.Trp486*) mis-localized diffusely to the cytoplasm (Fig. 3F), indicating loss of function effects.

**Fig. 4:**
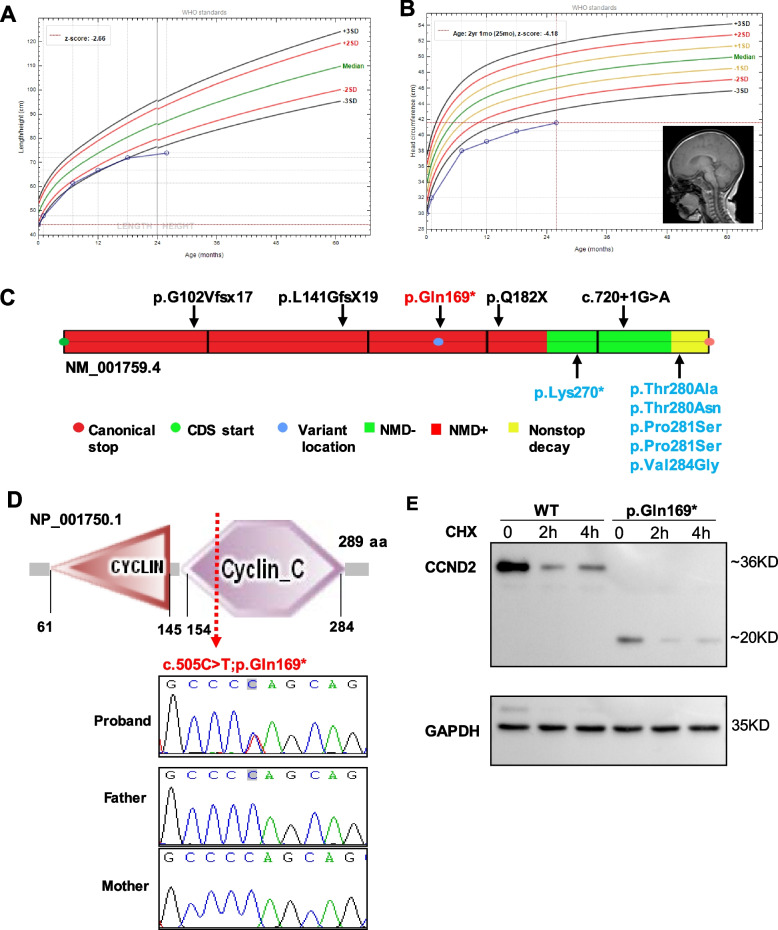
A loss-of-function *CCND2* variant in a female patient with primary microcephaly and short stature. **A**, Growth charts tracking the height measurements of patient NJ3099 on female growth curve. **B**, Head circumference measurements on growth curve and brain MRI of NJ3099. **C**, Schematic representation of the *CCND2* gene showing the localization of truncating variants that subjecting (NMD +) and escaping (NMD-) to nonsense-mediated RNA decay regions (https://nmdpredictions.shinyapps.io/shiny/)*.* Six previously reported gain of function variants in patients with megalencephaly-polymicrogyria-polydactyly-hydrocephalus syndrome are shown in blue. The loss-of-function variant p.Gln169* detected in this study is shown in red. The previously published four *CCND2* loss of function variants are shown in black. The region of *CCND2* where truncating variants trigger NMD is indicated in red. The region that escape NMD are represented in green. The nonstop decay region is indicated in yellow. **D**, Depicts the protein structure of CCND2, which contains two cyclin-like domains. Sequencing chromatograms of the heterozygous de novo* CCND2* variant in the proband and WT sequence detected in the parents. The index family’s heterozygous *CCND2* variant c.505C > T leads to a premature stop codon resulting in p.Gln169Ter. **E**, Transient overexpression of N-terminally Flag-tagged cDNA constructs modeling the wild-type allele and mutant CCND2 (p.Gln169Ter) in HEK293 cells. Protein extracts from transfected cells treated with cycloheximide (CHX) at time point 0, 2 h and 4 h were analyzed by western blotting with an antibody to Flag. The p.Gln169Ter variant produced a lower mount band with decreased expression and showed a drop in protein levels after inhibition of protein translation by CHX

Patient NJ3099 showed microcephaly (30 cm, -3.27SD) and hypothyroidism at birth. Her brain MRI have shown no structural abnormalities except for overall small brain size. At her most recent follow-up at 2 years of age, she presented with short stature (height 74.0 cm, < -4SD), progressive microcephaly (41.6 cm, <  − 4SD), global developmental delay and hypothyroidism (Fig. 4A-B). Trio-based WES analysis identified a de novo nonsense variant in *CCND2* (NM_001759: c.505C > T;p.Gln169Ter, Fig. 4D). Putative *CCND2* loss of function variants have been previously reported as a strong microcephaly candidate gene in four unrelated patients [16, 17]. The variant p.Gln169Ter was likely to be disease causing as a loss of function effect because (1) the variant is predicted to cause loss of function, because the resulting mRNA transcript is likely to subjected nonsense-mediated decay (Fig. 4C); (2) it is exceedingly rare and absent from the the gnomAD database; (3) the *CCND2* locus exhibits high loss-of-function (LoF) intolerance (gnomAD probability for loss-of-function intolerance pLI = 0.99; (4) Homozygotes for a targeted null ccnd2 variant in mice showed decreased cerebellar granule cell and stellate neuron populations; (5) The mutant CCND2 (p.Gln169Ter) was subjected to proteasomal degradation in vitro compared to wild-type CCND2 (Fig. 4E).

### Novel candidates (*DOCK9*, *RHOF*) related to CDC42/RAC signaling related actin cytoskeletal organization

*DOCK9* encodes a guanine nucleotide-exchange factor that plays important roles in dendrite growth in hippocampal neurons through activation of the Rho GTPases Cdc42 [18]. In family NJ233, we identified compound heterozygous variants (NM_015296: c.5887C > T;p.Arg1963Trp/c.4849 T > C;p.Trp1617Arg) in *DOCK9* (Table 2 and Supplementary Fig. S2). The patient showed microcephaly, facial dysmorphism including hypertelorism and a flat nasal bridge, congenital esotropia, delayed development of speech and language, motor developmental delay and epilepsy. The brain MRI revealed dilated ventricles and simplified gyral pattern. The two variants were extremely rare in the gnomAD database and yielded predominantly deleterious prediction scores by three algorithms (PolyPhen-2, MutationTaster and SIFT). Two variants both located within the DHR2 domain of the protein and well conserved from *Homo sapiens* to *D. rerio* (Supplementary Fig. S2).

*RHOF* encodes a plasma membrane-associated small GTPase, which works cooperatively with CDC42 and Rac to generate additional structures, increasing the diversity of actin-based morphology [19, 20]. In family NJ2639, we identified compound heterozygous variants (NM_019034: c.142 T > C;p.Tyr48His/c.343C > A;p.Pro115Thr) in *RHOF* in a patient presenting with microcephaly, global developmental delay, motor developmental delay and hypotonia (Table 2 and Supplementary Fig. S2). The two variants were extremely rare in the gnomAD database and yielded predominantly deleterious prediction scores by three algorithms (PolyPhen-2, MutationTaster and SIFT). The two variants both located within the RHO domain of the protein and well conserved from *Homo sapiens* to *D. rerio* (Supplementary Fig. S2).

### Novel candidates (*ELAVL3*, *PPP1R9B*, *KCNH3*) involved in neuronal differentiation and maintenance

Neuronal Elav-like proteins are RNA-binding proteins that regulate RNA stability and alternative splicing, promote the differentiation and maturation of neurons [21]. *ELAVL3* is highly expressed in the adult brain. A de novo missense variant (NM_001420: c.889G > A;p.Glu297Lys) in *ELAVL3* was identified in patient NJ463 with severe cortical dysplasia, thin corpus callosum, dilated lateral ventricles, simplified gyral pattern and overlapping cranial sutures (Table 2 and Supplementary Fig. S2).

*PPP1R9B* (protein phosphatase 1, regulatory (inhibitory) subunit 9B) encodes spinophilin, a protein located in the heads of neuron dendritic spines [22]. Spinophillin functions as a targeting and regulatory subunit of protein phosphatase 1, an enzyme involved in postsynaptic signal integration, and has an essential modulatory function for synaptic transmission and dendritic spine morphology in mouse [22]. A highly conserved de novo missense variant (NM_032595: c.1610C > T;p.Ala537Val) was identified in patient NJ2544 who presented with microcephaly, facial dysmorphism, global developmental delay, motor developmental delay and atrial septal defect with pseudoventricular aneurysm (Table 2 and Supplementary Fig. S2). Notably, *PPP1R9B* was suggested as the driven gene for the neurologic phenotype of 17q21.33 microduplication [23, 24].

*KCNH3* (Potassium Voltage-Gated Channel Subfamily H Member 3) encodes a voltage-gated potassium channel alpha subunit, which is predominantly expressed in the forebrain [25]. Mice homozygous for a knock-out allele exhibit neuron hyperexcitability and epilepsy [26]. In mature neurons, *KCNH3* have been identified as a FOXG1-target gene and might involve in the FOXG1 syndrome pathology [27]. In patient NJ233 who presented with global developmental delay, microcephaly, growth retardation and slightly dysmorphic features was found to have compound heterozygous variants (NM_012284: c.961C > T;p.His321Tyr/c.2812_2813del;p.Leu938ValfsTer73) in *KCNH3* (Table 2 and Supplementary Fig. S2). However, it is important to note that although the candidacy of *KCNH3 w*as supported by its relevant biological functions, there are some disputable issues that should be addressed through additional functional study and more matched patients. Firstly, potassium channels are mainly causative for autosomal dominant disorders, such as KCNH1, KCND3. Secondly, the expression data from single-cell transcriptomic data of the developing mouse neocortex is moderate comparing with the other candidates identified in this study (Fig. 2A). Lastly, the missense variant p.His321Tyr has yielded moderate deleterious prediction scores by two algorithms (PolyPhen-2 and SIFT). Additional functional studies are needed to verify the loss of function effect.

### Additional mutated cases of candidate genes from 5066 families with NDDs

When querying our data repository for additional likely disease-causing variants in the eight candidate genes in 5066 families with NDDs, we identified two cases with variants in *DOCK9* and *PPP1R9B*, respectively (Supplementary Table 3). In particular, Individual NJ4386 with dysmorphic features, speech and language delay, autism and epilepsy carried compound heterozygous missense variant (c.898G > T;p.Asp300Tyr and c.5288G > A;p.Arg1763Gln) in *DOCK9*. The second individual (NJ2637) presented with microcephaly (42 cm, -2SD, at 1 years old), global developmental delay and growth delay. WES identified a heterozygous frameshift variant (c.1424_1425insA; p.Asp475GlufsTer8) in *PPP1R9B*, parental samples were unavailable for segregation analysis (Supplementary Table S3).

## Discussion

We here evaluated disease-causing variants or candidate genes in an unselected cohort of 103 patients with microcephaly. Totally, in 65 families (63.1%), causative SVs (P/LP) and clinically relevant CNVs were identified by an informatics pipeline, analyses tools and followed by Sanger validation and segregation studies. By analyses of the mutated genes for temporal-spatial expression pattern during mammalian cerebral cortex development, neurologic phenotype from existing animal models, pathway analysis of gene products and functional validations in cultured cells, we further prioritized one newly reported gene (*CCND2*) and seven convincing candidate genes that were not previously reported in regard to NDDs or microcephaly.

Two similar genomic studies in exclusive microcephaly cohorts have been published recently. The first study was focused on the genomic and phenotypic delineation of congenital microcephaly from mainly consanguineous families, showing that pathogenic variants in microcephaly primary hereditary (MCPH) genes were encountered in 24% of patients with congenital microcephaly [11]. According to OMIM, 28 genes are classified as *MCPH* genes. However, this category only accounts for 2.9% (3/103, two *ASPM* families and one *SASS6* family) of our cohort. We report a large number of patients (72.7%) with de novo variants in established neurodevelopmental phenotypes in humans. And, transcriptional regulation was the most frequently affected pathway in both primary and postnatal microcephaly group. Another recently published study by Boonsawat et.al applied WES and high-resolution chromosomal microarray analysis in 62 patients with primary and secondary microcephaly and identified causative variants in 48.4% of cases, along with candidate genes in another 25.8% [10]. The mutated known genes and supposed candidates from these three independent genomic studies shows little overlapping, indicating that microcephaly is highly heterogeneous genetically.

Although CNV analysis of the WES data is still being optimized for clinical usage, we integrated currently available XHMM analysis into our WES analysis pipeline and identified a pathogenic or likely pathogenic CNV in 15 families (16/103, 15.7%) in our cohort, supporting a significant role of CNVs in congenital and postnatal microcephaly etiology. In conclusion, WES is an effective tool for identifying pathogenic SNVs, INDELs, and CNVs simultaneously in neurodevelopmental disorder genes and provides further analysis of the families without variants for novel gene discovery.

*PWP2* is a high-level candidate for primary microcephaly. This gene involved in nucleolar processing of pre-18S ribosomal RNA [28, 29]. Depleting Pwp2 in the yeast Saccharomyces cerevisiae leads to defects in cytokinesis [15]. A study of *PWP2* homologue (pwp2h) during zebrafish development provided evidence that Pwp2h is present in highly proliferative regions, including the forebrain ventricular zone and endoderm-derived organs in the early larval stage [30]. Boglev et.al identified a zebrafish mutant with a recessive lethal variant in pwp2h, exhibiting smaller eyes, a smaller, misshapen head [31]. In pwp2h null larvae, the growth of the endodermal organs, eyes, brain, and craniofacial structures is severely arrested [31]. Thus, *PWP2* appears to be required for the cell cycle progression during development of the central nervous system, and the loss-of-function variants identified in our patient probably serve as the genetic basis of the neurodevelopmental defects.

De novo* CCND2* variants leading to stabilization of cyclin D2 are known to cause megalencephaly-polymicrogyria-polydactyly-hydrocephalus syndrome (MPPH), which is a rare megalencephaly syndrome [32]. Contrast to the previously reported MPPH associated variants with a gain of function effect, the early stop gain c.505C > T;p.Gln169Ter variant identified in this study is predicted to induce nonsense-mediated mRNA decay, and was subjected to proteasomal degradation in vitro. Knockout studies of the homologous gene in mouse suggest the essential roles of this gene in neurogenesis [33]. A study of ccnd2 knockout mice showed decreased cerebellar granule cell and stellate neuron populations in homozygous mutants [34]. This is the third reported association between loss of function CCND2 variant and microcephaly, to our knowledge. This case confirmed that the loss of function variants in CCND2 are responsible for a malformative microcephaly syndrome with growth failure in humans.

Among mutated genes of interest, de novo truncating variant in *IRF2BP1* serves as a candidate for developmental epileptic encephalopathy. This gene encodes interferon regulatory factor-2-binding protein-1. In this study, we described a patient who carry damaging de novo nonsense variant (c.136G > T;p.Glu46Ter) in *IRF2BP1* and was affected with neonatal-onset microcephaly, epilepsy, hypotonia and global developmental delay. *IRF2BP1* and its two mammalian paralogs (*IRF2BPL* and *IRF2BP2*) share two highly conserved domains [35]: these included the N-terminal zinc finger domain for DNA binding domain and a C3HC4-type RING finger domain at the carboxyl terminus (Supplementary Fig. S2). The IRF2BP proteins has been proposed to enable transcription corepressor activity and ubiquitin protein ligase activity [36]. In addition, IRF2BP1 and its binding partners IRF2BP2 and IRF2BPL has also recently been suggested to control early transcription in EGFR signaling [37]. Notably, de novo truncating variants in IRF2BPL are lately reported to cause developmental epileptic encephalopathy [38].

In this study, we identified promising candidates that exhibit notable enrichment during early embryonic or fetal stages. Among these, *PWP2, CCND2, IRF2BP1*, and *ELAVL3* show the highest expression during early embryonic development, and patients with mutations in these genes present with congenital reduced brain volume. This finding aligns with the well-established understanding that primary microcephaly often arises from impaired neurogenesis and loss of neural progenitor cells [39]. Moreover, our gene–gene interaction analysis disclosed that our candidate genes interact with one another and with the known mutated genes in our cohort through co-expression (49.62%) and physical interactions (28.08%). In-depth examination of this network enables the identification of potentially involved biological processes, such as *CCND2*, which exhibits the strongest physical interactions with CDK6 (Cyclin-dependent kinase 6) and CDK4 (Cyclin-dependent kinase 4), suggesting its role in regulating the cell cycle. We have identified eight novel candidate genes that may be associated with primary and secondary microcephaly. It is crucial to emphasize that replicating these findings in a larger patient cohort is necessary for establishing a robust genotype–phenotype correlation. Furthermore, functional studies would be invaluable in evaluating these candidate genes.

## Conclusions

We established a high diagnostic yield (63.1%) for microcephaly in an unselected Chinese cohort and reported seven novel neurodevelopmental disorders /microcephaly candidate genes, which involved in cell cycle and division (*PWP2)*, CDC42/RAC signaling related actin cytoskeletal organization (*DOCK9, RHOF*), neurogenesis (*ELAVL3, PPP1R9B*) and transcription regulation (*IRF2BP1*). We confirmed that loss of function variants in *CCND2* associated with microcephaly, short stature, and developmental delay. Reporting potential candidate genes from clinical genomics accelerates the identification of morbid genome of novel monogenic neuro-disorders, serving as a foundation for evaluation and genetic counseling of the highly heterogeneous group of neurodevelopmental disorders.

## Materials and methods

### Study participants

From a total of 5066 in house exome data repository (2016–2021), we included 103 patients with microcephaly as one of observed features in this study. All patients were referred to us by their pediatricians, and had undergone WES as one of their genetic testing. We retrospectively included patients who met the following criteria: OFC > 2 SDs below the mean at birth or later, based on World Health Organization (WHO) and established growth charts; (2) no clear evidence for an acquired etiology or history of perinatal infection; and (3) without an unequivocal etiological diagnosis after clinical assessment by pediatricians. The clinical data provided by referring pediatrician were standardized to Human Phenotype Ontology (HPO) terms. Written informed consent (data repository, further research purposes for genes not yet associated to human diseases and scientific publication) was given by patients, parents, or referring physicians. This study was approved by the ethics committee of Children's Hospital of Nanjing Medical University.

### Whole exome sequencing and variant calling and filtering

WES was performed as previously described [40]. In brief, genomic DNA was isolated from blood lymphocytes and subjected to exome capture using the xGen Exome Research Panel v1.0 probe sequence capture array from IDT (Integrated Device Technology, USA), followed by next-generation sequencing on the Illumina novaseq (Illumina, USA) platform. Low-quality variations of the quality score < 20 (Q20) were filtered out. Sequencing reads were mapped to the GRCh37/Hg19 reference genome via Burrows-Wheeler Aligner (BWA) software. Filtering was performed to retain only alleles with a minor allele frequency (MAF) < 0.1%. MAF was estimated using combined datasets incorporating all available data from the 1,000 Genomes Project, the Exome Variant Server (EVS) project, dbSNP145, and gnomAD. Variants were confirmed by Sanger sequencing and for segregation of phenotype with genotype.

### Screening for variants in known genes with established Mendelian diseases and CNVs

**Fig. 5:**
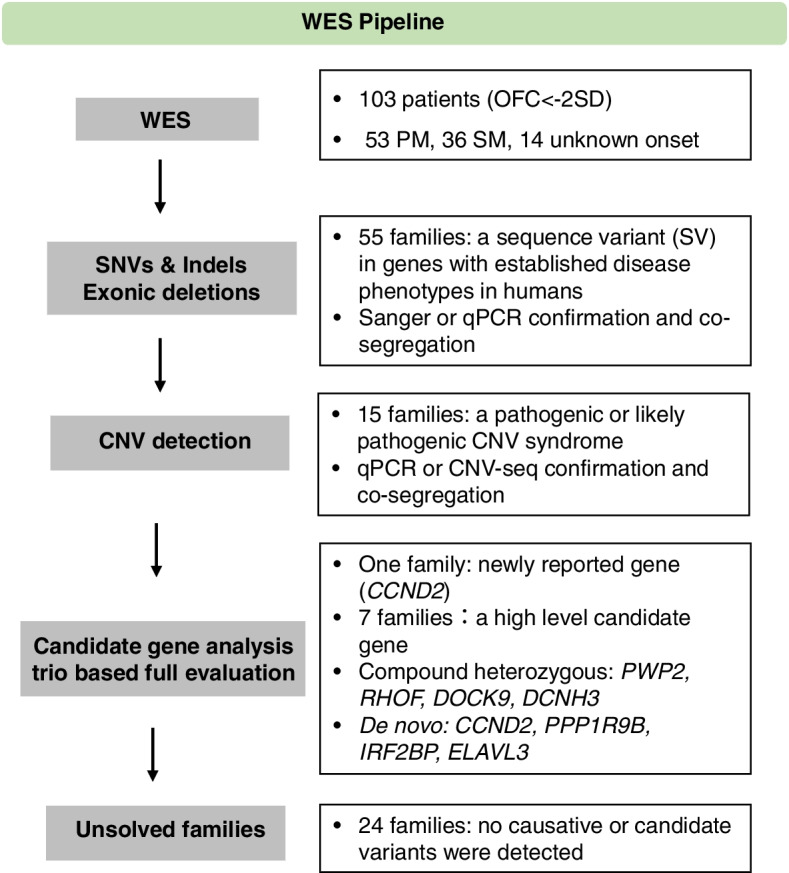
Overall workflow of our WES pipeline. CNV, copy-number variation; Indel, insertion/deletion; PM, primary microcephaly; SM, secondary microcephaly; SNV, single-nucleotide variant; WES, whole-exome sequencing

We performed WES of 19,396 genes in humans, covering Mendelian disorders included in the OMIM up to December 2021. We analyzed WES data using an in-house tool (http://cloud.chigene.org/) from Chigene (Beijing, China), Translational Medical Research Center Co. Ltd. Our workflow is shown in Fig. 5. Filtering was performed to retain only alleles with a minor allele frequency (MAF) < 0.1%. MAF was estimated using combined datasets incorporating all available data from the 1,000 Genomes Project, the Exome Variant Server (EVS) project, gnomAD and in house control set. Variant analysis was performed by geneticists and cell biologists, who had knowledge regarding clinical phenotypes, pedigree structure, genetic mapping, and in line with proposed American College of Medical Genetics and Genomics (ACMG) guidelines. The identified sequence variants (SVs) in solved cases were confirmed by Sanger sequencing and for segregation of phenotype with genotype, and were classified according to the American College of Medical Genetics and Genomics (ACMG) guidelines [41]. Each step in the SVs discovery and the median number of SVs requiring geneticists’ analysis are provided in Supplementary Fig. S3.

CNVs calling was performed using the same parameters and commands as previously described [42]. Briefly, we calculated coverage depth using analysis-ready BAM files generated from the BWA/Picard/GATK data-processing pipeline, following GATK best practices (https://gatk.broadinstitute.org/hc/en-us/sections/360007226651-Best-Practices-Workflows). For each exon target in each sample, the coverage depth values were averaged over their range to generate a raw read depth matrix for the target sample. Subsequently, filter out extremely variable and extreme targets [43]. Finally, rare coding CNVs were classified according to technical standards for the interpretation and reporting of constitutional copy number variants from ACMG and clingene [44]. Each step in the CNV discovery and the median number of CNVs requiring geneticists’ analysis are provided in Supplementary Fig. S4.

### Trio based full evaluation to identify novel candidates

If no causative variants or CNV was found, an analysis towards identification of potential novel candidate genes for neurodevelopmental disorders was applied by trio based full evaluation including filtering of the single nucleotide variants and indels with segregated de novo, homozygous/compound heterozygous, or maternally inherited X linked variants.

### Consideration of structural data and evolutionary conservation for variant evaluation

Protein domain structure depictions and evaluation was based on the uniprot (Universal Protein Resource) database. Orthologous proteins used to evaluate evolutionary conservation were obtained from the Ensemble Genome Browser and were aligned using the Clustal Omega multiple sequence alignment tool (EMBL-EBI).

### Functional evaluations of variants in PWP2 and CCND2

Wildtype and mutant plasmid constructions, cell culture, transfection, cycloheximide (CHX) chase experiment, immunoblotting, immunofluorescence and imaging were performed to evaluate functional consequences of identified variants in PWP2 and CCND2 (Supplementary Methods).

### Web resources

Clustal Omega, http://www.ebi.ac.uk/Tools/msa/clustal

Ensembl Genome Browser, http://www.ensembl.org

Genome Aggregation Database (gnomAD), http://gnomad.broadinstitute.org

GeneMANIA, http://genemania.org/

HGMD, https://portal.biobase-international.com/hgmd

Human Phenotype Ontology, https://hpo.jax.org/app/

Mouse Genome Informatics, http://www.informatics.jax.org/

MutationTaster, http://www.mutationtaster.org

Nmdpredictions, https://nmdpredictions.shinyapps.io/shiny/

Online Mendelian Inheritance in Man (OMIM), http://www.omim.org

Polyphen2, http://genetics.bwh.harvard.edu/pph2

Sorting Intolerant from Tolerant (SIFT), http://sift.jcvi.org

Uniprot Consortium, http://www.uniprot.org/

Single cell portal, https://singlecell.broadinstitute.org/single_cell

## Supplementary Information

**Additional file 1: Supplementary Table S1. **Detailed phenotypic and genotypic data of 55 cases with a monogenic cause.

**Additional file 2: Supplementary Figure S1. **The expressional trajectories in neocortex and annotation of the mutated known genes in the cohort; **Supplementary Figure S2. **Sanger sequencing confirmation and segregation of the identified novel candidate genes; **Supplementary Figure S3. **Flowchart of calling SVs from exome sequence data using GATK, and each step in the SVs annotating and filtering is listed; **Supplementary Figure S4. **Flowchart of calling CNV from exome sequence data using XHMM; **Supplementary table S2. **The CNV identified from WES data in 15 patients in our cohort; **Supplementary table S3. **Additional mutated cases of candidate genes from 5066 families with NDDs; **Supplementary methods. Figure S1. **The expressional trajectories in neocortex and annotation of the mutated known genes in the cohort. **Figure S2.** Sanger sequencing confirmation and segregation of the identified novel candidate genes. **Figure S3.** Flowchart of calling SVs from exome sequence data using GATK, and each step in the SVs annotating and filtering is listed. **Figure S4.** Flowchart of calling CNV from exome sequence data using XHMM. **Figure 4D.** Transient overexpression of N-terminally Flag-tagged cDNA constructs modeling the wild-type allele and two independent PWP2 variants (p.Arg660Gln and p.Trp486*) in HEK293 cells. **Figure 5E.** Transient overexpression of N-terminally Flag-tagged cDNA constructs modeling the wild-type allele and mutant CCND2 (p.Gln169*) in HEK293 cells. **Table S1.** **Table S2.** The CNV identified from WES data in 15 patients in our cohort. **Table S3.** Additional mutated cases of candidate genes from 5066 families with NDDs. 

## Data Availability

All data generated or analysed during this study are included in this published article and its supplementary information files.

## Abbreviations

NDDs: Neurodevelopmental disorders
WES: Whole exome sequencing
SVs: Sequence variants
CNVs: Copy number variants
OFC: Occipitalfrontal circumference
PM: Primary microcephaly
SM: Secondary microcephaly
WHO: World Health Organization
HPO: Human Phenotype Ontology
IDT: Integrated Device Technology
BWA: Burrows-Wheeler Aligner
MAF: Minor allele frequency
EVS: Exome Variant Server
ACMG: American College of Medical Genetics and Genomics
CHX: Cycloheximide
LoF: Loss-of-function
MCPH: Microcephaly primary hereditary
MPPH: Megalencephaly-polymicrogyria-polydactyly-hydrocephalus
