# Supplementary material for: Diagnostic yield and novel candidate genes for neurodevelopmental disorders by exome sequencing in an unselected cohort with microcephaly
Source: BMC Genomics. 2023 Jul 27;24:422. doi: 10.1186/s12864-023-09505-z (PMC10373276; doi:10.1186/s12864-023-09505-z)
Supplement: Supplementary file 2 — Additional file 2: Supplementary Figure S1. The expressional trajectories in neocortex and annotation of the mutated known genes in the cohort; Supplementary Figure S2. Sanger sequencing confirmation and segregation of the identified novel candidate genes; Supplementary Figure S3. Flowchart of calling SVs from exome sequence data using GATK, and each step in the SVs annotating and filtering is listed; Supplementary Figure S4. Flowchart of calling CNV from exome sequence data using XHMM; Supplementary table S2. The CNV identified from WES data in 15 patients in our cohort; Supplementary table S3. Additional mutated cases of candidate genes from 5066 families with NDDs; Supplementary methods. Figure S1. The expressional trajectories in neocortex and annotation of the mutated known genes in the cohort. Figure S2. Sanger sequencing confirmation and segregation of the identified novel candidate genes. Figure S3. Flowchart of calling SVs from exome sequence data using GATK, and each step in the SVs annotating and filtering is listed. Figure S4. Flowchart of calling CNV from exome sequence data using XHMM. Figure 4D. Transient overexpression of N-terminally Flag-tagged cDNA constructs modeling the wild-type allele and two independent PWP2 variants (p.Arg660Gln and p.Trp486*) in HEK293 cells. Figure 5E. Transient overexpression of N-terminally Flag-tagged cDNA constructs modeling the wild-type allele and mutant CCND2 (p.Gln169*) in HEK293 cells. Table S1. Table S2. The CNV identified from WES data in 15 patients in our cohort. Table S3. Additional mutated cases of candidate genes from 5066 families with NDDs. [file 12864_2023_9505_MOESM2_ESM.pdf]

Supplementary Data

Supplementary Figure S1. The expressional trajectories in neocortex and annotation of the mutated known genes in the cohort

Supplementary Figure S2. Sanger sequencing confirmation and segregation of the identified novel candidate genes

Supplementary Figure S3. Flowchart of calling SVs from exome sequence data using GATK, and each step in the SVs annotating and filtering is listed.

Supplementary Figure S4. Flowchart of calling CNV from exome sequence data using XHMM.

Supplementary table S2. The CNV identified from WES data in 15 patients in our cohort

Supplementary table S3. Additional mutated cases of candidate genes from 5066 families with NDDs

Supplementary methods

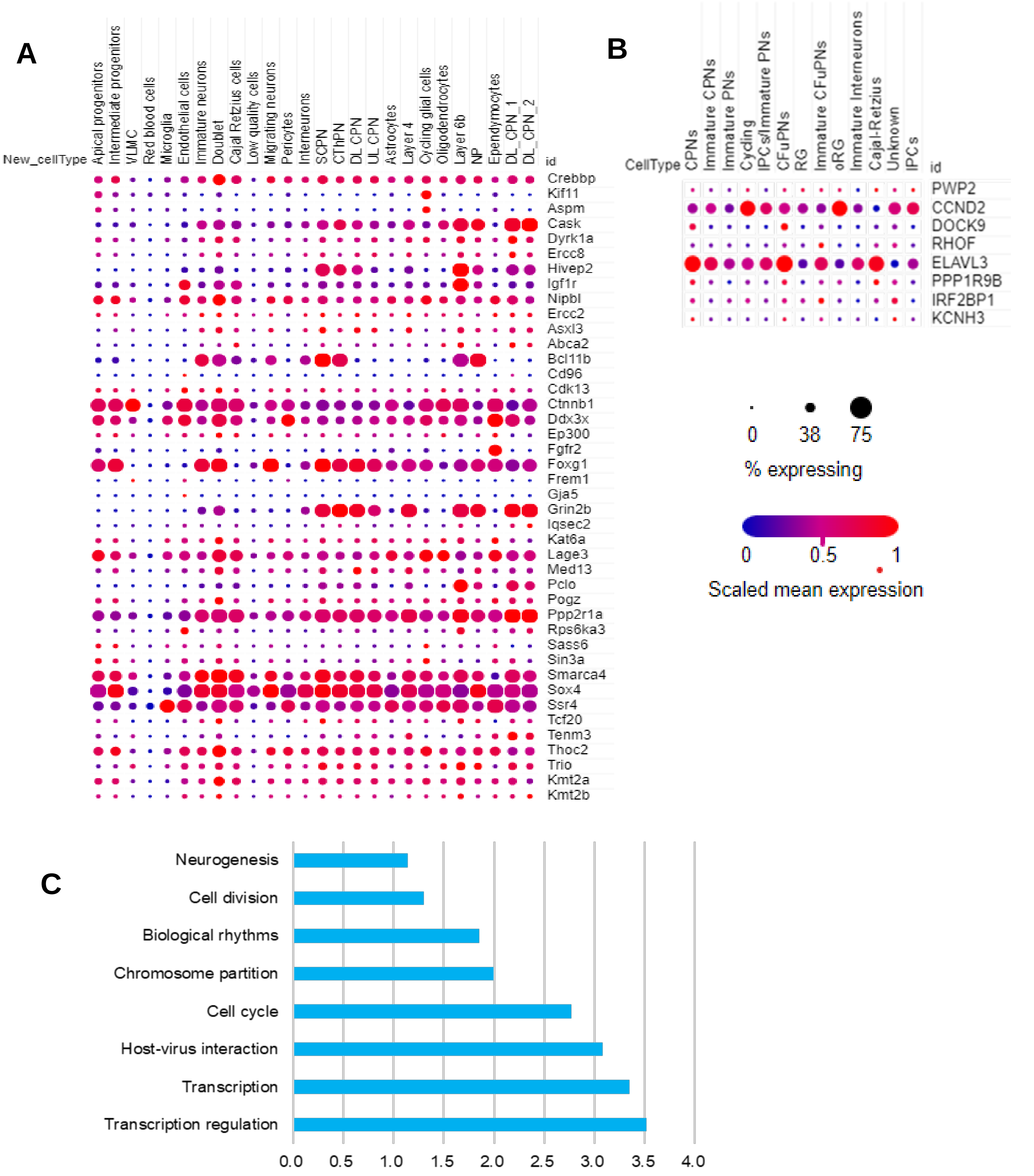

Supplementary Figure S1. A, The expressional trajectories of the mutated known genes in cerebral cortex from published single-cell mRNA sequencing data ( ); B, The expressional trajectories of 8 candidate genes in human brain organoids derived from stem cells (data from Paola Arlotta et.al *Nature.* 2019,570(7762):523-527); C, Biological functional annotation of the novel and known mutated genes in our cohort.

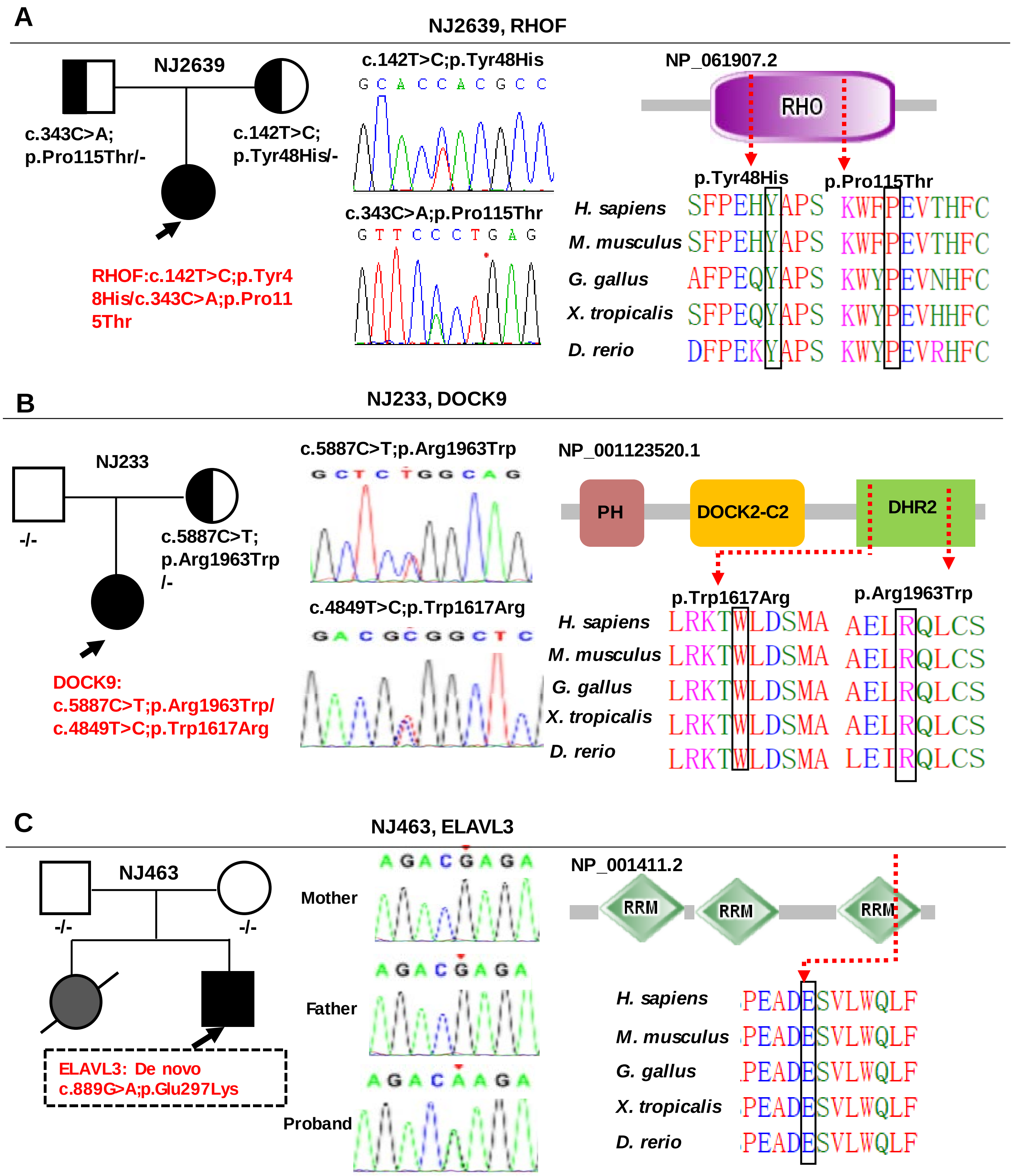

Supplementary Figure 2.

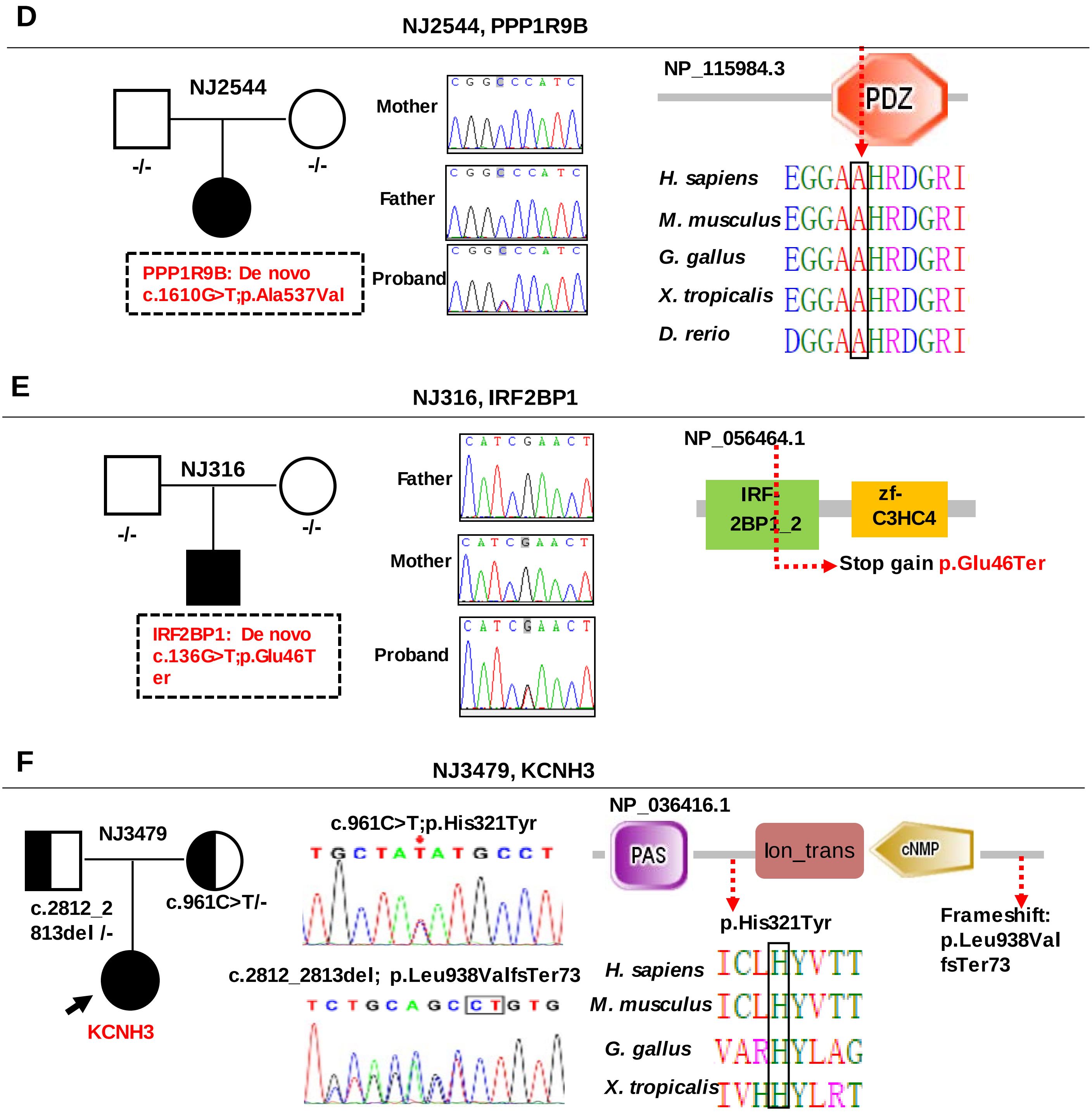

Supplementary Figure 2.

Supplementary Figure S2. Sanger sequencing confirmation and segregation of the identified novel candidate genes

A， In family NJ2639, we identified compound heterozygous variants (NM_019034: c.142T>C;p.Tyr48His/c.343C>A;.Pro115Thr) in *RHOF* in a patient presenting with microcephaly, global developmental delay, motor developmental delay and hypotonia。

B, In family NJ233, we identified compound heterozygous variants (NM_015296: c.5887C>T;p.Arg1963Trp/c.4849T>C;p.Trp1617Arg) in *DOCK9. The* patient presented with microcephaly, facial dysmorphism including hypertelorism and a flat nasal bridge, congenital esotropia, delayed development of speech and language, motor developmental delay and epilepsy.

C, In family NJ463, a de novo missense variant (NM_001420: c.889G>A;p.Glu297Lys) in *ELAVL3* was identified. The patient showed severe cortical dysplasia, thin corpus callosum, dilated lateral ventricles, simplified gyral pattern and overlapping cranial sutures.

D, In family NJ2544, a highly conserved de novo missense variant (NM_032595: c.1610C>T;p.Ala537Val) was identified in the proband who presented with microcephaly, facial dysmorphism, global developmental delay, motor developmental delay and atrial septal defect with pseudoventricular aneurysm.

E, Patient NJ316 carried a damaging de novo nonsense variant (c.136G>T;p.Glu46Ter) in IRF2BP1 and was affected with neonatal-onset microcephaly, epilepsy, hypotonia and global developmental delay.

F, In patient NJ233 who presented with global developmental delay, microcephaly growth retardation and slightly dysmorphic features was found to have compound heterozygous variants (NM_012284: c.961C>T;p.His321Tyr/ c.2812_2813del; p.Leu938ValfsTer73) in KCNH3.

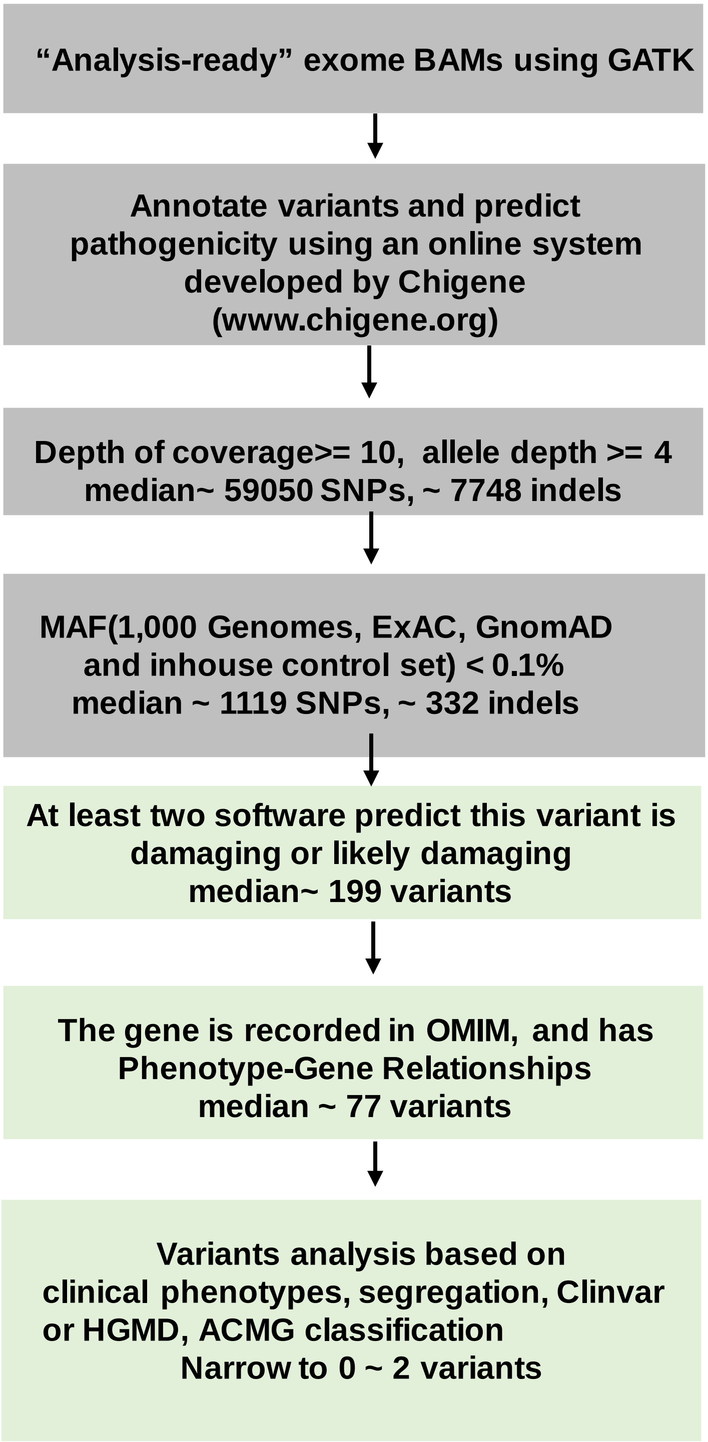

Supplementary Figure S3 Flowchart of calling SVs from exome sequence data using GATK, and each step in the SVs annotating and filtering is listed. The green blocks indicate the steps of variant filtering and interpretation that require analysis by geneticists.

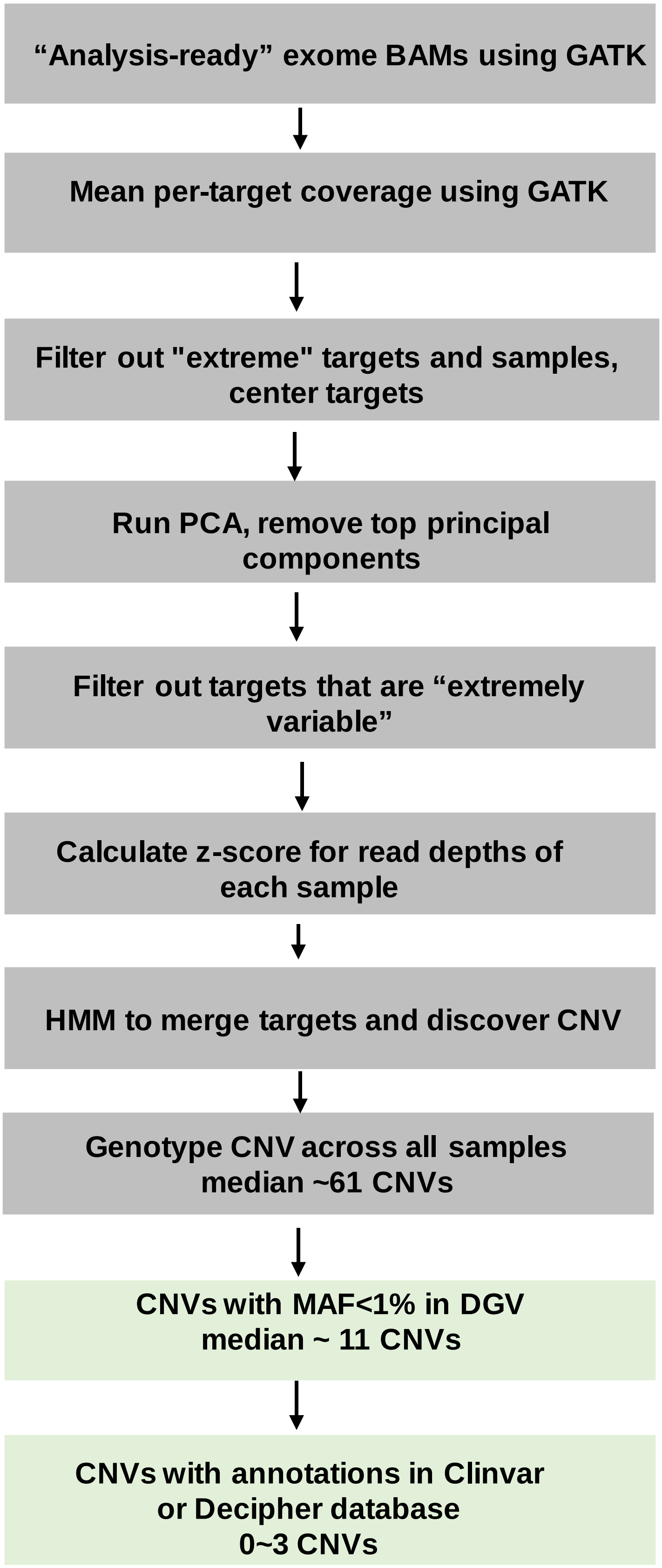

Supplementary Figure S4 Flowchart of calling CNV from exome sequence data using XHMM. Each step in the CNV discovery and genotyping XHMM pipeline is listed. The green blocks indicate the steps of variant filtering and interpretation that require analysis by geneticists.

Supplementary table S2. The CNV identified from WES data in 15 patients in our cohort

Family	Sex	Age	Phenotype (HPO term)	PM or SM or Unknown	CNV syndrome	chr;loss/gain/ Mb	ACMG	Segregation maternal; paternal (other)
NJ1139	F	4m	High palate, microcephaly, short stature, weight loss, poor head control	SM	Skraban-Deardorff syndrome	chr1:223949282-231837770:gain2; 7.89Mb	P	de novo
NJ957	M	1m	Bilateral cryptorchidism, small penis, small scrotum, microcephaly, small jaw , abnormal morphology of the face, limb hypertonia, increased circulating procalcitonin level, patent ductus arteriosus, cardiac murmur, thrombocytopenia, edema, fever, elevated C-reactive protein level, pneumonia, shortness of breath, neonatal asphyxia, neonatal sepsis, hepatomegaly	Unknown	NA	chr3:183639087-189038609:loss1; 5.4Mb	P	de novo
NJ1132	M	5y	Microcephaly, increased distance between eyes, poor social interactions, delayed speech and language development, attention deficit hyperactivity disorder, cognitive impairment	Unknown	Early-onset Alzheimer disease with cerebral amyloid angiopathy	chr21:13200000-48129895:gain1;34.9Mb	P	unknown
NJ1520	M	10m	Microcephaly, agenesis of corpus callosum, delayed myelination, abnormality of lateral ventricle, motor developmental delay, cognitive impairment, hypothyroidism, adducted thumb, atrial septal defect, patent foramen	PM	15q26 overgrowth syndrome	chr13:107145387-115091756:loss1; 7.95Mb and chr15:100514606-	P; VUS	de novo

			ovale,abnormality of the tricuspid valve ,pulmonary arterial hypertension,valgus foot deformity			102359328:gain1; 1.84Mb		
NJ2563	M	8m	Microcephaly, bushy eyebrows, global developmental delay, muscle hypertonia, abnormality of body weight, reduced subcutaneous adipose tissue, abnormal pulmonary vein morphology, malnutrition	PM	Prader-Willi syndrome/Angelman syndrome(15q11.2 deletion)	chr15:22833524-23086411:loss1;252.89 Kb	P	unknown
NJ330	M	2y	Microcephaly, pruritus, hyperbilirubinemia, hyperlipidemia, increased total bilirubin, increased serum bile acid concentration, elevated cholesterol ester level,hepatomegaly, cholestatic liver disease,elevated hepatic transaminase	SM	Alagille syndrome	chr20:9965785-11725865:loss1;1.76Mb	P	F
NJ2225	M	2y	Microcephaly, low anterior hairline,abnormality of the outer ear, poor social interactions, delayed speech and language development, global developmental delay, sleep disturbance, attention deficit hyperactivity disorder, motor developmental delay, eczema	SM	Prader-Willi syndrome/Angelman syndrome	chr15:1-102531392:upd1; 102.53Mb	P	unknown
XT2341	M	1y	Small penis, microcephaly, samll jaw,global developmental delay, malnutrition, short stature, patent ductus arteriosus, abnormality of the anterior fontanelle,abnormality of upper lip ,abnormal teeth spacing	PM	NA	chr7:98983338-100860555:loss1; 1.877Mb	LP	unknown
XT2699	F	1m	Global developmental delay, atrial septal defect, anal atresia, microcephaly, external ear malformation,low-set ears,increased distance between eyes, small jaw, high palate, polydactyly, simian line,muscle hypotonia	PM	22q11 deletion syndrome	chr22:18894077-21652015:loss1;2.757Mb	P	unknown

NJ1536	F	7y	Microcephaly, intellectual disability, attention deficit hyperactivity disorder	unknown	Cri du Chat Syndrome	chr5:10461295-17276009:loss1;6.81Mb	P	unknown
NJ2972	M	1y	Microcephaly, strabismus,macrotia, global developmental delay, motor developmental delay, hypothyroidism,abnormality of the thyroid gland	SM	NA	chr13:30341391-43987050:loss1;13.65Mb	P	unknown
NJ3904	M	3y	Global developmental delay, microcephaly, short stature	Unknown	Xq28 Microduplication	chrX:153577216-153846529:gain;269.31Kb	P	unknown
NJ3371	F	6y4m	Intellectual disability, microcephaly, autism	PM	22q13 deletion syndrome (Phelan-Mcdermid syndrome)	chr22:50832337-51220722:loss1;388.39Kb	P	unknown
NJ3293	M	7y	Intellectual disability, microcephaly, abnormal facial shape, attention deficit hyperactivity disorder, aortic valve stenosis, dental anomalies	SM	Williams-Beuren Syndrome (WBS)	chr7:72717592-74251505:loss1;1.53Mb	P	unknown
NJ3156	M	5y	Intellectual disability, microcephaly, attention deficit hyperactivity disorder, delayed speech and language development, feeding difficulties, carious teeth, epicanthus	SM	Prader-Willi syndrome/Angelman syndrome	chr15:23605426-28632839:loss1;5.03Mb	P	unknown
NJ2321	M	7m	Global developmental delay, microcephaly, intracranial hemorrhage, abnormal posturing, premature birth,aortic valve stenosis, ventricular septal defect	SM	NA	chr7:147600656-158935237:loss1;11.33Mb	P	de novo

Supplementary table S3. Additional mutated cases of candidate genes from 5066 families with NDDs

Family	Sex	Age	Phenotype (HPO term)	Gene	Zygosity	Variant (hg19;c.;p.)	Polyphen CADD Mutationtaster	gnomAD	Reference transcript	Segregation maternal; paternal (other)
NJ4386	Female	2y11m	Autism, attention deficit hyperactivity disorder, delayed speech and language, developmentincreased distance between eyes, epicanthus	*DOCK9*	Het	chr13:99461688;c.5288G>A;p.Arg1763Gln	0.867;24.0;D	0/8/278374	NM_015296	maternal
				DOCK9	Het	chr13:99566647;c.898G>T;p.Asp300Tyr	0.129;26.0;D	0/1/236968	NM_015296	paternal
NJ2637	female	1y	Microcephaly, global developmental delay, growth delay	*PPP1R9B*	Het	c.1424_1425insA; p.Asp475GlufsTer8	Frameshift	/	NM_032595	Unknown

Supplementary Methods

Construction of plasmid vector

cDNA of human PWP2 and CCND2 were cloned into the pcDNA3.1-3×Flag vector using the Clon Express Entry One Step Cloning Kit (Vazyme, China). The corresponding mutations were introduced with the PCR-based DpnI-treatment method using the Mut Express II Fast Mutagenesis Kit V2 (Vazyme, China) and appropriate primers. The entire coding sequences of all the constructs were verified using sequencing.

Cell culture and transfection

HEK293 cells were transiently transfected with wild-type or mutant constructs. Briefly, HEK293 cells were seeded in 6-well plates with 2 mL of Dulbecco's Modified Eagle's Medium (DMEM) in each well at 37°C in an atmosphere of 5% CO_2 24 h prior to transfection. After the cells were 50%-70% confluent, HEK293 cells were transfected with purified constructs using PolyJet™ DNA In Vitro Transfection Reagent (SignaGen, American). Transfection was performed for 4-6 h with 2 μg of constructs. For cycloheximide (CHX) chase experiments, cells were treated with 25 μM CHX (Sigma-Aldrich C4859-1ML) 24 hours after transfection at different time point.

Immunofluorescence and laser scanning confocal microscopy

The transfected HEK293 cells were fixed with acetone for 20 min at 4 ºC and rinsed with PBS and incubated with mouse anti-Flag (1:400, Sigma Aldrich) overnight at 4 ºC. The secondary antibodies were added and incubated for 2 h at 37 ºC. Imaging was performed on an inverted confocal laser scanning microscope using a 63/1.4 oil immersion objective.

Immunoblot analysis

HEK293 cells were rapidly washed with ice-cold PBS, and whole cell lysates were generated using lysis buffer containing protease inhibitors. The protein concentration was determined using a Micro BCA protein assay kit with bovine serum albumin as the standard (Pierce, Thermo). Total protein (20 μg)

was separated using 10% SDS-PAGE and transferred onto PVDF membranes. The membranes were blocked in TBST (0.1% Tween 20 in TBS) containing 5% nonfat milk for 1 h at room temperature and incubated with primary antibodies against Flag (1: 3000, Sigma Aldrich) and GAPDH (1: 3000, ProTech) overnight at 4°C. The membranes were incubated with HRP-labeled secondary antibodies at room temperature for 1 h, and protein bands were visualized using a chemiluminescence reaction.
